# A Digital Health Framework to Assess Glycemia and Physical Activity in Kidney Transplant Candidates: A Pilot Study

**DOI:** 10.1097/TXD.0000000000001910

**Published:** 2026-01-20

**Authors:** Carina M. Flaherty, Christopher Sanchez, Celina Liu, Dhairya Upadhyay, Dorry L. Segev, Nicole Ali, Joseph Lee, Mara McAdams-DeMarco, Morgan E. Grams, Souptik Barua

**Affiliations:** 1 Division of Precision Medicine, Department of Medicine, New York University Grossman School of Medicine, New York, NY.; 2 Department of Surgery, New York University Grossman School of Medicine, New York, NY.; 3 Department of Population Health, New York University Grossman School of Medicine, New York, NY.; 4 Transplant Institute, New York University Grossman School of Medicine, New York, NY.

## Abstract

**Background.:**

Kidney transplant recipients are at risk for adverse health outcomes. Digital health tools such as wearable accelerometers and continuous glucose monitors (CGMs) can provide detailed, noninvasive tracking of health behaviors and measures, such as physical activity, sleep, and glucose levels, that may offer insights into future health concerns, such as posttransplant diabetes mellitus, cognitive health, and transplant rejection. However, there is limited evidence on the feasibility and acceptability of these devices in kidney transplant candidates older than 50 y.

**Methods.:**

This observational cross-sectional pilot study aimed to examine the feasibility of 2 digital health tools: an accelerometer and a continuous glucose monitor. Participants were eligible for the study if they were living donor kidney transplant candidates, aged 50 y or older, had no known cognitive impairments, and could provide informed consent. Participants were asked to wear a CGM and an accelerometer for up to 14 d before their kidney transplant surgery. Device feasibility was quantified by (1) the total time the devices were worn, and (2) the validated System Usability Scale survey administered after the devices were returned.

**Results.:**

20 participants enrolled in the study (mean age 64 ± 9 y, 25% women, 40% with type 2 diabetes). The median number of days of accelerometer and CGM wear were 7 (interquartile range, 6–10) d and 7 (interquartile range, 7–10) d, respectively. Ninety percent of participants reported a favorable opinion of both devices. Participants wore the CGM 100% of the time and the accelerometer 90% of the time, indicating high adherence.

**Conclusions.:**

The use of digital devices was acceptable among kidney transplant candidates aged older than 50 y, paving the way for larger studies to identify early digital biomarkers of health outcomes in this high-risk population.

## Graphical abstract

**Figure:**
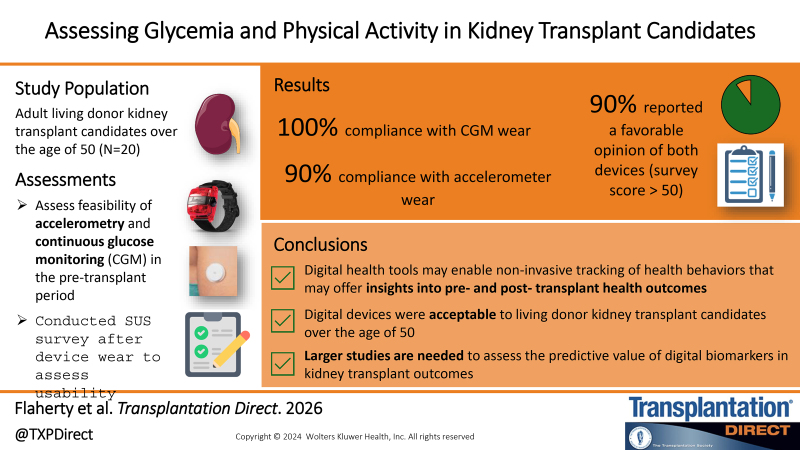

## INTRODUCTION

Every year, >130 000 individuals in the United States develop end-stage kidney disease (ESKD), the majority of whom are adults older than 50 y.^1^ Kidney transplantation is widely viewed as the optimal treatment for most patients with ESKD, increasing survival and quality of life while reducing medical expenditures compared with other disease management options, such as dialysis.^2^ Recent work has pushed to expand the age threshold for transplantation, and since 2015, transplant rates in patients aged 50 y or older have increased dramatically.^3^ Although it can be the best treatment option for ESKD, kidney transplant recipients (KTRs) are at increased risk for numerous health outcomes and face complex medical management care after transplant. KTRs can face adverse health outcomes such as posttransplant diabetes mellitus (PTDM),^4^ accelerated cognitive decline,^5,6^ allograft dysfunction,^7^ and cardiometabolic complications.^8^ These challenges can be exacerbated for transplant recipients as they age, as older recipient age has been shown to be an independent risk factor for the development of many of these outcomes,^4^ emphasizing the need for new solutions and innovative research aimed at mitigating these risks.

Emerging digital health tools offer new opportunities to enhance disease management and preventative measures as they can be used to monitor health metrics, provide personalized feedback, and support lifestyle modifications before and after transplant. Furthermore, data from wearable devices can be used for predictive modeling of clinical outcomes,^9^ which is especially important in the kidney transplant population, which is at higher risk of health complications after transplant. Hyperglycemia, low physical activity, and sleep disorders are prevalent in KTRs and are associated with an increased risk of adverse outcomes such as graft loss,^10^ cardiovascular disease^11^ and mortality.^12^ Two digital health tools that may be used to provide continuous health observation in KTRs are accelerometry and continuous glucose monitoring (CGM). Accelerometry devices can be used to measure physical activity and sleep patterns and have been previously used in studies of KTRs^13,14^ to assess the impact on kidney function markers and to study demographic and clinical correlates. CGM devices are used to record and track glucose levels during wear, and preliminary studies have shown their utility for understanding PTDM.^15^ In this study, we sought to assess the acceptability and feasibility of simultaneously using an accelerometer and a continuous glucose monitor in adults older than 50 y scheduled to receive a living donor kidney transplant, with the goal of adding to the literature on how these devices may provide insights into predicting and preventing adverse health outcomes and posttransplant complications in this population.

## MATERIALS AND METHODS

This pilot study enrolled patients seen at the NYU Langone Health Transplant Institute in New York City who were aged 50 y and older and scheduled for a living donor kidney transplant with NYU Langone Health. The study team focused on living donor transplant candidates since the timing of transplantation is unpredictable for deceased donor kidney transplant surgery and would impede data collection before transplant. Study participants needed to be willing to wear the devices and capable of providing informed consent.

This study used the Abbott Freestyle Libre Pro CGM and the Actigraph GT3X accelerometer to assess glucose and physical activity/sleep, respectively. Previous studies have analyzed the Abbott Freestyle Libre CGM and Actigraph GT3X accelerometer in individuals with ESKD: Studies with the Freestyle Libre Pro CGM have analyzed the accuracy of the device in individuals with chronic kidney disease and undergoing hemodialysis treatment, including those with type 1 diabetes^16^, with type 2 diabetes,^16-18^ and in those without diabetes.^18^ The Actigraph GT3X accelerometer has been used to assess physical activity in KTRs^14^ and in individuals on hemodialysis.^19,20^ NYU Langone Health Transplant Institute clinicians on the study team introduced the study to patients, and interested and eligible patients were contacted by the study team for consent and enrollment. Enrollment took place before transplant, either remotely or during a pretransplant clinic visit at the Transplant Institute. The study team asked participants to wear the accelerometer and CGM from enrollment through the day of transplant surgery, or up to 14 d, whichever came first. Participants were asked to return the devices to the study team by mail or at a future clinic visit. Participants were compensated $40 for wearing each of the devices. Following device wear, participants were sent the System Usability Scale (SUS) survey,^21^ a 10-item Likert scale survey, to complete for both the accelerometer and the CGM. Participants completed the survey either remotely or in person at a posttransplant clinic visit with a member of the study team. Participants were compensated $20 for completing the survey.

The primary outcome for this study was feasibility. To analyze the primary outcome, descriptive statistics including mean, SD, median, and interquartile range (IQR) were calculated for the number of days of CGM wear, and the number of days with at least 10 h of accelerometer wear during the day and accelerometer wear during sleep. Participant-reported usability of the CGM and accelerometer devices was analyzed using SUS scores, and descriptive statistics were calculated for both devices. Secondary outcomes included average glucose levels, glucose variability, and time in range measures from the CGM, as well as average daily step count, sedentary time, moderate-to-vigorous physical activity (MVPA), and sleep duration derived from accelerometer data in the Actilife software using the Freedson 1998 calibrations^22^ for activity classification, and the Cole-Kripke scoring^23^ for sleep detection. To reliably estimate the main sleep event of a day, fragmented sleep events reported by the Actilife software were merged into one sleep period if the gap between successive sleep events was <1 h. To prevent biased estimates due to shorter sleep events, such as naps, only the longest sleep event >4 h^24^ was used to estimate daily sleep duration statistics. For days with >70% valid CGM data, >10 h of accelerometer wear, or a detected major sleep episode, standard time-series analysis methods were applied. All statistical analyses were conducted using R or Python software, with a significance level set at *P* value of <0.05 and results reported with 95% confidence intervals where applicable.

This study was reviewed and approved by the NYU Langone Health Institutional Review Board (No. i23-00942) in October of 2023. The study was conducted in accordance with the Code of Federal Regulations on the Protection of Human Subjects (45 CFR Part 46), any other applicable US government research regulations, and institutional research policies and procedures. Informed consent was obtained before the initiation of any study procedures.

## RESULTS

Of the 20 participants enrolled, 25% self-identified as women, 25% as Hispanic or Latino, 40% had type 2 diabetes, and 55% were on dialysis (age, 64 ± 9 y; body mass index, 29 ± 5 kg/m^2^; Table 1). Seventeen participants wore both devices, whereas 3 participants wore the accelerometer only. The duration of wear for the CGM device was a median 7 (IQR, 7–10) d (Table 2; **Figure S1, SDC,** https://links.lww.com/TXD/A826). Sixteen of 17 participants (94%) had complete CGM data during their prescribed wear period, indicating minimal noncompliance. The median SUS score was 73 (IQR, 64–84), and 94% of participants had a net positive opinion of the CGM (SUS >50; Table 2; **Figure S2, SDC,** https://links.lww.com/TXD/A826).

**TABLE 1.** Baseline characteristics

Characteristics	N = 20, n (%)
Age, y, mean (SD)	64 (9)
Female, n (%)	5 (25)
Self-reported race, n (%)	
White	14 (70)
Black	2 (10)
Asian	2 (10)
Not specified	2 (10)
Self-reported ethnicity, n (%)	
Non-Hispanic or Latino	16 (80)
Hispanic or Latino	4 (20)
Self-reported household income, n (%)	
<$50 000	2 (10)
$50 000–$100 000	4 (20)
$100 000–$150 000	2 (10)
>$150 000	8 (40)
Prefer not to answer	4 (20)
Self-reported highest level of education, n (%)	
High school degree or equivalent	4 (20)
Some college or Associate’s degree	3 (15)
Bachelor’s degree	3 (15)
Master’s degree	6 (30)
Doctorate degree	4 (20)
Type 2 diabetes before transplant, n (%)	8 (40)
On dialysis before transplant, n (%)	11 (55)
BMI, kg/m^2^, mean (SD)	28.9 (5)

BMI, body mass index.

**TABLE 2.** CGM (n = 17) and accelerometry (n = 20) outcomes

Outcome	
Continuous glucose monitor	
N	17
Days of CGM wear, median (IQR)	7 (7–10)
System Usability Scale Survey Score, median (IQR)	73 (64–84)
Glucose level, mg/dL, median (IQR)	101 (91–115)
Glucose variability, median (IQR)	28.6% (19.0%–32.2%)
Percent time spent in euglycemia range (70–140 mg/dL), median (IQR)	74% (50%–93%)
Percent time spent in hyperglycemia (>180 mg/dL), median (IQR)	0.8% (0%–5.1%)
Accelerometry	
N	20
Daytime days of accelerometer wear, median (IQR)	7 (6–10)
Nighttime days of accelerometer wear, median (IQR)	6 (4–7)
System Usability Scale Survey Score, median (IQR)	73 (56–81)
Daily step count, median (IQR)	6414 (4320–8312)
Daily moderate-to-vigorous physical activity time, min, median (IQR)	72 (38–99)
Daily sedentary time, h, median (IQR)	13 (11–15)
Sleep duration, h, median (IQR)	7.6 (7.0–9.7)

CGM, continuous glucose monitor; IQR, interquartile range.

Among the 20 participants who wore the accelerometer device, the median number of days with at least 10 h of accelerometer wear was 7 (IQR, 6–10) d, and the median number of nights in which the accelerometer was worn during sleep was 6 (IQR, 4–7; Table 2; **Figure S1, SDC,** https://links.lww.com/TXD/A826). As a percentage of the wear period, 90% (IQR, 76–100) of the days of wear had at least 10 h of activity data, indicating a small degree of noncompliance during the prescribed wear period. The median SUS score for the accelerometer was 73 (IQR, 56–81), with 95% of the participants having a net positive opinion of the accelerometer (SUS >50; Table 2; **Figure S2, SDC,** https://links.lww.com/TXD/A826).

The median glucose level in our cohort (n = 17) was 101 mg/dL (IQR, 91–115 mg/dL). Glucose variability measured as the coefficient of variation was 28.6% (IQR, 19.0–32.2). The time spent in the euglycemia range (70–140 mg/dL) was 74% (IQR, 50–93), with minimal time spent by the participants in hyperglycemia (>180 mg/dL) of 0.8 (IQR, 0–5.1). The median daily step count was 6414 (IQR, 4320–8312). Fourteen of 20 participants (70%) got at least 5000 steps per day. The median daily MVPA was 72 (IQR, 38–99) min, with 16 of 20 participants (80%) getting the recommended minimum 30 min of daily MVPA. The median daily sedentary time was 13 (IQR, 11–15) h. The median sleep duration was 7.6 (IQR, 7.0–9.7) h; with 14 of 20 participants (70%) getting the recommended 7 h of daily sleep (Table 2).

## DISCUSSION

Wearable devices such as accelerometers and CGMs have the potential to transform pre- and posttransplant care for kidney transplant candidates and recipients as they age. By providing continuous, real-time monitoring of physical activity, sleep patterns, and glucose levels pre- and posttransplant, these tools may enable early detection of adverse health outcomes, including PTDM, cognitive decline, and allograft dysfunction, and support proactive management of health behaviors and lifestyle after transplant. The use of continuous data from wearable devices may enhance peritransplant management. For example, future studies might integrate CGM data in the early posttransplant period to model the risk of PTDM, enabling applications such as optimized titration of immunosuppressive medications and dietary counseling to minimize postprandial hyperglycemia. Sufficient physical activity in the peritransplant period has been shown to improve graft function and reduce frailty; therefore, accelerometry data may help optimize physical activity counseling in kidney transplant candidates and recipients. This study has several strengths: Participants were highly adherent to device wear and return, and survey responses reflected positive participant feedback. However, limitations include a small sample size, a short observation period, the possibility that incentives influenced adherence rates, and a lack of long-term follow-up, although these limitations are expected for a pilot study. Because the cohort included only living donor kidney transplant candidates, the findings may not generalize to the deceased donor population, which is more racially and socioeconomically diverse and may face different feasibility challenges with digital health tools.^25,26^ Importantly, recent research highlights that reducing costs and implementing equity-focused interventions to improve access to CGM tools have led to promising uptake among underserved groups.^27,28^ This study focused on the feasibility of wearable devices pretransplant, recognizing that baseline glucose and activity levels pretransplant are crucial predictors of posttransplant outcomes. However, our feasibility metrics may not generalize to the early posttransplant phase, where patients’ health management is more complex and dynamic compared with pretransplant. Future studies are therefore needed to assess the usability of these devices posttransplant, which can provide valuable data on glycemia and activity patterns associated with posttransplant complications. Finally, there is evidence that the ActiGraph algorithms used to determine activity-intensity metrics may be less precise in adults older than 59 y, as validity can vary depending on accelerometer placement, the type of activity, and the specific parameter being assessed.^29^ Previous research has also indicated accelerometry-derived sleep may be less accurate in adults with disturbed sleep, which may impact sleep estimation in individuals with ESKD.^30^

Future research should focus on larger, longitudinal studies to evaluate the predictive value of digital biomarkers for adverse health outcomes in older KTRs and explore the usability and acceptance of these devices across diverse populations, including individuals with varying levels of digital literacy. As wearable technology becomes more widely adopted, integrating digital health data into kidney transplant care, along with additional data from the electronic health record, could support precision medicine-guided approaches to healthcare management and improve patient outcomes.

This pilot study highlights the feasibility and acceptability of wearable accelerometers and CGMs among adults aged older than 50 y preparing for living donor kidney transplantation. The findings underscore the potential of digital health tools to enhance pre- and posttransplant care and warrant further investigation. Additional research in this area could shed more light on how incorporating digital health technologies into routine clinical practice for kidney transplant candidates and recipients may play a significant role in mitigating risks and improving outcomes.

## Supplementary Material


