# Supplementary material for: A Digital Health Framework to Assess Glycemia and Physical Activity in Kidney Transplant Candidates: A Pilot Study
Source: Transplant Direct. 2026 Jan 20;12(2):e1910. doi: 10.1097/TXD.0000000000001910 (PMC12818855; doi:10.1097/TXD.0000000000001910)

**Online Supplementary Material**

**TITLE**: A Digital Health Framework to Assess Glycemia and Physical Activity in Kidney Transplant Recipients

**Figure S1:** Participant-level data on days of wear for the (a) continuous glucose monitor (n=17) and (b) accelerometer (n=20). For the accelerometer, we report both the number of days with at least 10 hour of accelerometer weartime (excluding sleep) and number of days with sleep data.

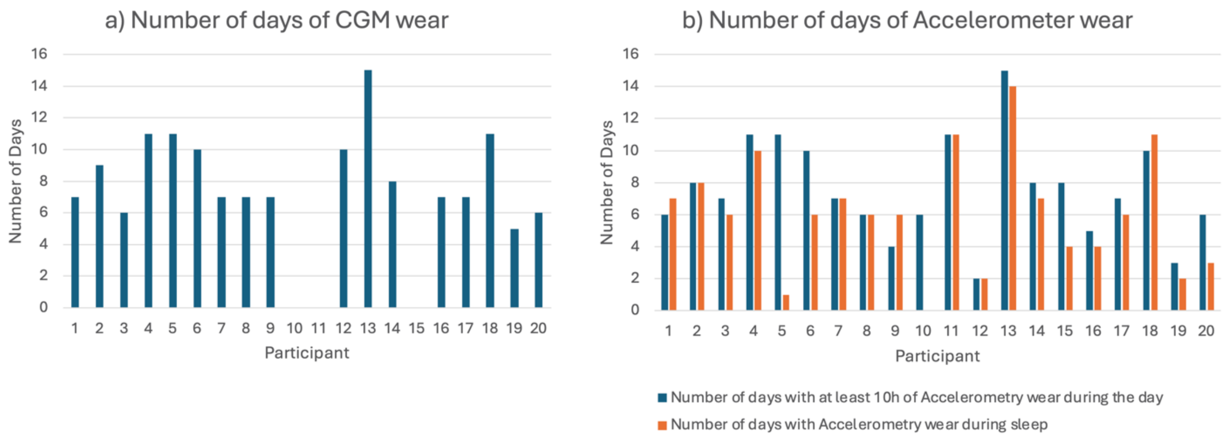


**Figure S2:** Participant-level system usability scale survey scores for the continuous glucose monitor (n=17) (blue) and accelerometer (n=20) (orange).

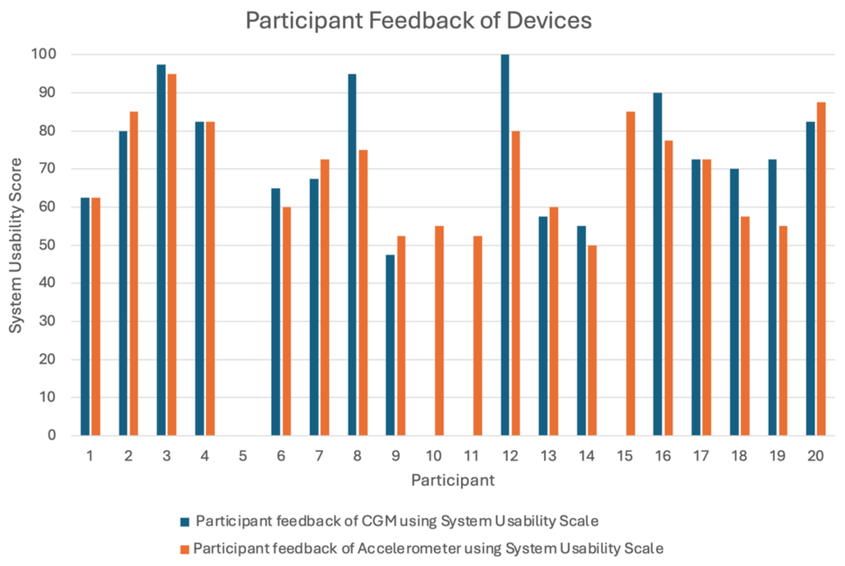

Supplement: Supplementary file 1 [file txd-12-e1910-s001.pdf]
